# Relationship between Urinary Phthalate and Bisphenol A Concentrations and Serum Thyroid Measures in U.S. Adults and Adolescents from the National Health and Nutrition Examination Survey (NHANES) 2007–2008

**DOI:** 10.1289/ehp.1103582

**Published:** 2011-07-11

**Authors:** John D. Meeker, Kelly K. Ferguson

**Affiliations:** Department of Environmental Health Sciences, University of Michigan School of Public Health, Ann Arbor, Michigan, USA

**Keywords:** biomarkers, epidemiology, exposure, population, risk

## Abstract

Background: Limited animal, *in vitro*, and human studies have reported that exposure to phthalates or bisphenol A (BPA) may affect thyroid signaling.

Objective: We explored the cross-sectional relationship between urinary concentrations of metabolites of di(2-ethylhexyl) phthalate (DEHP), dibutyl phthalate (DBP), and BPA with a panel of serum thyroid measures among a representative sample of U.S. adults and adolescents.

Methods: We analyzed data on urinary biomarkers of exposure to phthalates and BPA, serum thyroid measures, and important covariates from 1,346 adults (ages ≥ 20 years) and 329 adolescents (ages 12–19 years) from the National Health and Nutrition Examination Survey (NHANES) 2007–2008 using multivariable linear regression.

Results: Among adults, we observed significant inverse relationships between urinary DEHP metabolites and total thyroxine (T_4_), free T_4_, total triiodothyronine (T_3_), and thyroglobulin, and positive relationships with thyroid-stimulating hormone (TSH). The strongest and most consistent relationships involved total T_4_, where adjusted regression coefficients for quintiles of oxidative DEHP metabolites displayed monotonic dose-dependent decreases in total T_4_ (*p*-value for trend < 0.0001). Suggestive inverse relationships between urinary BPA and total T_4_ and TSH were also observed. Conversely, among adolescents, we observed significant positive relationships between DEHP metabolites and total T_3_. Mono(3-carboxypropyl) phthalate, a secondary metabolite of both DBP and di-*n*-octyl phthalate, was associated with several thyroid measures in both age groups, whereas other DBP metabolites were not associated with thyroid measures.

Conclusions: These results support previous reports of associations between phthalates—and possibly BPA—and altered thyroid hormones. More detailed studies are needed to determine the temporal relationships and potential clinical and public health implications of these associations.

Thyroid hormones play a vital role in many physiological processes including fetal and child growth and brain development, as well as energy balance, metabolism, and other functions in the nervous, cardiovascular, pulmonary, and reproductive systems of children and adults (Diamanti-Kandarakis et al. 2009; Miller et al. 2009). Recent studies of humans, animals, and *in vitro* screening assays provide evidence that exposures to chemicals commonly encountered in our environment, such as phthalates and bisphenol A (BPA), may affect thyroid signaling through a number of potential mechanisms (Boas et al. 2006; Zoeller 2007).

Phthalates and BPA are high-production chemicals used in a variety of applications related to consumer products, and exposure to these chemicals among the general population is widespread [Centers for Disease Control and Prevention (CDC) 2010]. The potential for these agents to alter thyroid signaling has recently gained increased attention. For example, recent studies have reported declines in measures of brain development in relation to phthalates (Cho et al. 2010; Engel et al. 2009, 2010; Kim et al. 2009) and BPA (Braun et al. 2009), and thyroid signaling has been hypothesized as one potential mechanism involved in these associations. Studies suggest that altered thyroid function may also be involved in relationships between these chemicals and increased waist circumference, insulin resistance, and diabetes among adults (Galloway et al. 2010; Hatch et al. 2010; Lang et al. 2008). However, limited human studies have investigated the relationship between exposure to phthalates or BPA and altered thyroid signaling. Mono(2-ethylhexyl) phthalate (MEHP), the urinary monoester metabolite of the plasticizer di(2-ethylhexyl) phthalate (DEHP), was inversely associated with serum free thyroxine (T_4_) and total triiodothyronine (T_3_) levels in a cross-sectional study of 408 men attending a U.S. infertility clinic (Meeker et al. 2007). Urinary DEHP metabolites were also inversely related to total and free T_3_ levels in a recent cross-sectional study of 845 Danish children (Boas et al. 2010). In animal studies, rats with diets contaminated with DEHP were found to have thyroid alterations and lower plasma T_4_ concentrations compared with controls (Hinton et al. 1986; Howarth et al. 2001; Poon et al. 1997; Price et al. 1988). In addition, a recent *in vitro* study reported that DEHP and other phthalates caused changes in the iodide uptake of thyroid follicular cells (Wenzel et al. 2005).

Dibutyl phthalate (DBP), a lower molecular weight phthalate used as a plasticizer and in adhesives, solvents, and certain personal care products, may also affect thyroid signaling. DBP metabolite concentrations in urine were inversely related to serum levels of total and free T_3_ in a study of Danish children (Boas et al. 2010) and inversely related to serum levels of total and free T_4_ in a Taiwanese study of 76 pregnant women (Huang et al. 2007). O’Connor et al. (2002) reported a dose- dependent inverse association between DBP and T_3_ and T_4_ levels in male rats. BPA, which is used in polycarbonate plastic, epoxy resins, and other products, has also been reported to exert effects on the thyroid receptor in experimental studies (Diamanti-Kandarakis et al. 2009; Wetherill et al. 2007). Human studies of BPA and thyroid hormones are lacking, although a suggestive inverse relationship was observed between urinary BPA concentrations and thyroid-stimulating hormone (TSH) in a cross-sectional study of 167 men from an infertility clinic (Meeker et al. 2010).

The present analysis was carried out to investigate whether urinary biomarkers of exposure to DEHP, DBP, and BPA were associated with thyroid function measures collected in the National Health and Nutrition Examination Survey (NHANES) 2007–2008, a stratified multistage probability sample of the civilian noninstitutionalized population of the United States.

## Methods

The present analysis included measurements from 2 years of NHANES data, 2007–2008. NHANES is an ongoing cross-sectional study designed to collect nationally representative data on dietary intake and disease. Methods for demographic and survey data collection are described in detail elsewhere [National Center for Health Statistics (NCHS) 2010b].

*Urinary phthalate metabolites and BPA.* Urine samples collected at a mobile examination center collection were stored at 4°C or frozen at –20°C and then shipped to the Division of Environmental Health Laboratory Sciences, CDC, for analysis. Measurement of urinary phthalate metabolite concentrations was conducted using online solid-phase extraction (SPE), isotope dilution, and high-performance liquid chromatography (HPLC) separation, followed by electrospray ionization and tandem mass spectrometry (MS/MS), as described in detail elsewhere (NCHS 2010d; Silva et al. 2004). Urinary BPA concentrations were measured using online SPE coupled to isotope dilution, HPLC, and atmospheric pressure chemical ionization–MS/MS (NCHS 2010c; Ye et al. 2005). Quality control procedures for all analytes followed those described by Westgard et al. (1981), and values below the limit of detection (LOD) were replaced with a value of the LOD divided by the square root of 2 (Hornung and Reed 1990).

In addition to urinary BPA, we focused our analyses on primary and secondary metabolites of DEHP [MEHP and three oxidized DEHP metabolites: mono(2-ethyl-5-hydroxyhexyl) phthalate (MEHHP), mono(2-ethyl-5-oxohexyl) phthalate (MEOHP), and mono(2-ethyl-5-carboxypentyl) phthalate (MECPP)] and DBP [mono-*n*-butyl phthalate (MnBP), mono-isobutyl phthalate (MiBP), and mono(3-carboxypropyl) phthalate (MCPP), an oxidized metabolite of both DBP and di-*n*-octyl phthalate (DOP)], because previous studies have suggested that exposure to these phthalates or their metabolites may be associated with altered thyroid hormones.

Because all urinary biomarker concentrations were right-skewed, the data were transformed using the natural logarithm (ln) prior to analysis. In addition, metabolite measurements were creatinine standardized for presentation of descriptive statistics and calculation of simple correlations by dividing metabolite concentrations by urinary creatinine (micrograms per gram creatinine). For regression analysis, we used unadjusted metabolite levels; urinary creatinine was included as a covariate in all models (Barr et al. 2005).

*Serum thyroid measures.* The NHANES thyroid panel, including measures of free and total T_3_ and T_4_, TSH, and thyroglobulin, was measured in serum collected at the same time as urine samples and analyzed using various immunoenzymatic assays as described elsewhere (NCHS 2009). Because of limited serum available for thyroid hormone analysis, this aspect of the study excluded all children under 12 years of age. All distributions except total T_3_ and total T_4_ were right-skewed and ln-transformed for analysis.

*Covariates.* Demographic data were collected in an in-home survey component of NHANES. From these data, we examined age, sex, race and ethnicity, and education level as potential confounding variables. From examination and laboratory data we considered body mass index (BMI), serum cotinine (log-transformed) as a measure of exposure to tobacco smoke, and urinary iodine (log-transformed). Variables were evaluated for inclusion by examining bivariate relationships with urinary biomarkers and serum thyroid measures and by their significance in full models. Variables that were significantly associated with one or more urinary biomarkers or serum thyroid measures or that were statistically significant (α = 0.05) in more than one multivariable model were included in the final models. All models were adjusted for the same covariates for consistency.

*Statistical analysis.* There were 2,035 subjects ≥ 12 years of age available who had data for one or more of the urinary phthalate metabolites, urinary BPA, urinary creatinine, and one or more of the thyroid measures. We excluded from our analysis 164 subjects with a reported history of thyroid disease, 20 women who were pregnant, and 88 subjects with data missing for age. We also excluded 3 subjects with outlying/influential values, which included 2 subjects with outlying high levels of free and total T_3_ and T_4_ and low levels of TSH, as well as 1 subject with outlying high levels of total T_4_. This resulted in 1,760 total subjects available for analysis. An additional 85 participants were missing data on covariates (14 missing BMI, 3 missing serum cotinine, and 68 missing urinary iodine) and were not included in the multivariable models, leaving 1,675 participants (1,346 adults and 329 adolescents 12–19 years of age).

Data analysis was performed using SAS 9.2 (SAS Institute Inc., Cary, NC). NHANES data are collected using a complex, multistage study design. For our analysis, we used 2-year weights for individual probabilities drawn from the urinary biomarker data sets according to the NCHS web tutorial (NCHS 2010a) to account for the sampling method. In addition, stratum and cluster weights were included in regression models to correct for the study design. For comparison, we also constructed models that did not include the sample weights, because the weighted method may result in an inefficient analysis due to the large variability in assigned weights, and because adjustments for variables used in the creation of weights (such as age, sex, and ethnicity) are already included in the full models (Korn and Graubard 1991).

In descriptive analyses, we explored differences in urinary biomarker or serum thyroid measures between categories using Wilcoxon rank-sum or Kruskal-Wallis tests. Spearman rank correlations were calculated to assess relationships between continuous variables. Because of significant differences in thyroid hormone levels in adolescents compared with adults in these data, we separated our data set into two groups (ages 12–19 years and > 20 years) in subsequent analyses.

We then constructed full multivariable linear regression models with serum thyroid measures as the dependent variable and individual ln-transformed urinary phthalate metabolite or BPA concentration as a predictor along with age (continuous variable), sex (dichotomous), race and ethnicity (categorical), ln-transformed serum cotinine (continuous), BMI (continuous), ln-transformed iodine (continuous), and ln-transformed urinary creatinine (continuous) as covariates. Analyses were performed both with and without including the sample weights to examine the effects of weighting. Secondary analyses were conducted by repeating all models when stratifying on sex. Regression results are presented as the change in thyroid measure associated with a unit increase in ln-transformed phthalate metabolite concentration. To improve interpretability, we also provide several examples in the “Results” where we back-transformed the regression coefficient to represent a change in hormone measure associated with an interquartile range (IQR) increase in urinary phthalate metabolite concentration. Finally, for urinary exposure biomarkers detected in > 80% of samples, we explored evidence for nonlinear relationships by assessing relationships between urinary phthalate metabolite or BPA quintiles and serum thyroid measures. Tests for trend were conducted for ordinal urinary biomarker quintiles in regression models using integer values (0–4).

## Results

Population characteristics and distributions of urinary phthalate metabolites, BPA, and serum thyroid measures are presented in Supplemental Material, Tables 1–3 (http://dx.doi.org/10.1289/ehp.1103582). As reported in previous NHANES investigations (Calafat et al. 2008; CDC 2010; Silva et al. 2004), we observed associations between urinary phthalates and/or BPA and age, race, sex, and BMI (data not shown). All of the urinary biomarkers included in the analysis were also positively and moderately correlated with urinary iodine, and positively but weakly correlated with serum cotinine (data not shown). Several thyroid measures were also associated with age, race, sex, BMI, cotinine, and urinary iodine (data not shown).

**Table 1:** Adjusted regression coefficients (95% CIs) for change in serum thyroid measure in relation to a unit increase in ln-transformed urinary phthalate or BPA concentration among adults (≥ 20 years of age; *n *= 1,346), with results unweighted for sampling strategy.^*a*^

Analyte	β (95% CI)	*p*-Value	β (95% CI)	*p-*Value	β (95% CI)	*p-*Value
		Total T_4_ (µg/mL)				ln-Free T_4_ (ng/dL)				ln-TSH (µIU/mL)		
MEHP		–0.14 (–0.22 to –0.071)		0.0001		–0.0039 (–0.012 to 0.0041)		0.34		–0.002 (–0.033 to 0.030)		0.92
MEHHP		–0.19 (–0.27 to –0.12)		< 0.0001		–0.0085 (–0.017 to –0.00035)		0.04		0.022 (–0.010 to 0.054)		0.18
MEOHP		–0.17 (–0.25 to –0.099)		< 0.0001		–0.0075 (–0.016 to 0.00072)		0.07		0.020 (–0.013 to 0.053)		0.23
MECPP		–0.19 (–0.27 to –0.11)		< 0.0001		–0.0071 (–0.016 to 0.0018)		0.12		0.020 (–0.015 to 0.055)		0.26
MiBP		0.020 (–0.075 to 0.11)		0.68		0.0010 (–0.0094 to 0.011)		0.86		–0.013 (–0.054 to 0.028)		0.52
MnBP		0.054 (–0.039 to 0.15)		0.26		0.0038 (–0.0065 to 0.014)		0.46		–0.022 (–0.062 to 0.019)		0.30
MCPP		–0.079 (–0.17 to 0.011)		0.08		–0.0022 (–0.012 to 0.0077)		0.66		0.009 (–0.030 to 0.048)		0.66
BPA		–0.095 (–0.19 to –0.00063)		0.049		0.0011 (–0.0093 to 0.011)		0.84		–0.032 (–0.073 to 0.0090)		0.13
		Total T_3_ (ng/dL)				ln-Free T_3_ (pg/mL)				ln-Thyroglobulin (ng/mL)		
MEHP		–0.78 (–1.83 to 0.28)		0.15		–0.0014 (–0.0063 to 0.0034)		0.56		–0.028 (–0.077 to 0.022)		0.27
MEHHP		–0.89 (–1.96 to 0.17)		0.10		–0.0027 (–0.0071 to 0.0027)		0.38		–0.027 (–0.077 to 0.023)		0.29
MEOHP		–0.96 (–2.04 to 0.12)		0.08		–0.0030 (–0.0079 to 0.0020)		0.24		–0.031 (–0.081 to 0.020)		0.23
MECPP		–0.56 (–1.73 to 0.61)		0.34		–0.0004 (–0.0057 to 0.0050)		0.89		–0.022 (–0.076 to 0.033)		0.44
MiBP		0.77 (–0.59 to 2.12)		0.27		–0.0012 (–0.0074 to 0.0051)		0.72		–0.018 (–0.081 to 0.045)		0.58
MnBP		1.25 (–0.10 to 2.59)		0.07		0.0002 (–0.0059 to 0.0064)		0.94		–0.021 (–0.084 to 0.042)		0.51
MCPP		–0.44 (–1.73 to 0.85)		0.51		–0.0072 (–0.013 to –0.0013)		0.02		–0.007 (–0.067 to 0.053)		0.82
BPA		–0.84 (–2.19 to 0.52)		0.23		–0.0002 (–0.0064 to 0.0060)		0.95		–0.028 (–0.092 to 0.035)		0.38
**a**Adjusted for age, sex, race, BMI, ln-serum cotinine, ln-urinary creatinine, and ln-urinary iodine; 10 participants were missing data for BMI, 1 for serum cotinine, and 48 for urinary iodine.												

**Table 2:** Adjusted regression coefficients (95% CIs) for change in serum thyroid measure in relation to a unit increase in ln-transformed urinary phthalate or BPA concentration among adults (≥ 20 years of age; *n *= 1,346), with results weighted for sampling strategy.^*a*^

Analyte	β (95% CI)	*p-V*alue	β (95% CI)	*p-*Value	β (95% CI)	*p-*Value
		Total T_4_ (µg/mL)				ln-Free T_4_ (ng/dL)				ln-TSH (µIU/mL)		
MEHP		–0.13 (–0.22 to –0.045)		0.006		–0.0054 (–0.020 to 0.0091)		0.44		0.028 (–0.0013 to 0.057)		0.06
MEHHP		–0.21 (–0.32 to –0.11)		0.0006		–0.010 (–0.027 to 0.0066)		0.22		0.046 (0.012 to 0.081)		0.01
MEOHP		–0.19 (–0.36 to –0.086)		0.001		–0.0088 (–0.026 to 0.0081)		0.28		0.047 (0.012 to 0.082)		0.01
MECPP		–0.23 (–0.33 to –0.13)		0.0002		–0.011 (–0.027 to 0.0057)		0.19		0.041 (0.0057 to 0.077)		0.03
MiBP		0.0003 (–0.11 to 0.11)		0.99		0.0005 (–0.011 to 0.012)		0.93		0.010 (–0.057 to 0.078)		0.75
MnBP		0.018 (–0.12 to 0.15)		0.78		0.0056 (–0.013 to 0.024)		0.54		–0.015 (–0.077 to 0.047)		0.62
MCPP		–0.088 (–0.20 to 0.029)		0.13		0.0007 (–0.015 to 0.016)		0.92		0.007 (–0.030 to 0.044)		0.70
BPA		–0.056 (–0.12 to 0.0071)		0.08		0.0036 (–0.010 to 0.017)		0.59		0.001 (–0.051 to 0.054)		0.96
		Total T_3_ (ng/dL)				ln-Free T_3_ (pg/mL)				ln-Thyroglobulin (ng/mL)		
MEHP		–0.83 (–2.17 to 0.051)		0.21		–0.00003 (–0.0085 to 0.0085)		0.99		–0.041 (–0.085 to 0.0025)		0.06
MEHHP		–1.49 (–2.72 to –0.26)		0.02		–0.0021 (–0.0082 to 0.0040)		0.47		–0.057 (–0.099 to –0.015)		0.01
MEOHP		–1.36 (–2.54 to –0.18)		0.03		–0.0024 (–0.0082 to 0.0035)		0.40		–0.058 (–0.099 to –0.018)		0.008
MECPP		–1.37 (–2.89 to 0.15)		0.07		0.0004 (–0.0063 to 0.0070)		0.91		–0.054 (–0.092 to –0.016)		0.009
MiBP		0.30 (–1.84 to 2.42)		0.77		–0.0041 (–0.011 to 0.0031)		0.25		–0.013 (–0.12 to 0.094)		0.80
MnBP		1.03 (–1.66 to 3.71)		0.43		–0.0019 (–0.0082 to 0.0044)		0.53		–0.021 (–0.095 to 0.053)		0.56
MCPP		–0.97 (–2.59 to 0.65)		0.22		–0.0079 (–0.014 to –0.0023)		0.009		–0.035 (–0.12 to 0.045)		0.36
BPA		–0.94 (–2.45 to 0.56)		0.20		0.0021 (–0.0079 to 0.012)		0.66		–0.030 (–0.092 to 0.031)		0.31
**a**Adjusted for age, sex, race, BMI, ln-serum cotinine, ln-urinary creatinine, and ln-urinary iodine; 10 participants were missing data for BMI, 1 for serum cotinine, and 48 for urinary iodine.												

**Table 3:** Adjusted regression coefficients (95% CIs) for change in serum thyroid measure in relation to a unit increase in ln-transformed urinary phthalate or BPA concentration among adolescents (12–19 years of age; *n *= 329), with results unweighted for sampling strategy.

Analyte	β (95% CI)	*p-*Value	β (95% CI)	*p-*Value	β (95% CI)	*p-*Value
		Total T_4_ (µg/mL)				ln-Free T_4_ (ng/dL)				ln-TSH (µIU/mL)		
MEHP		0.050 (–0.093 to 0.19)		0.49		–0.0042 (–0.018 to 0.0096)		0.55		0.012 (–0.039 to 0.063)		0.64
MEHHP		0.044 (–0.10 to 0.19)		0.55		–0.0029 (–0.017 to 0.011)		0.68		0.064 (0.013 to 0.12)		0.01
MEOHP		0.045 (–0.10 to 0.19)		0.55		–0.0037 (–0.018 to 0.011)		0.61		0.067 (0.015 to 0.12)		0.01
MECPP		0.085 (–0.081 to 0.25)		0.32		–0.0024 (–0.018 to 0.014)		0.77		0.070 (0.012 to 0.13)		0.02
MiBP		–0.034 (–0.25 to 0.19)		0.76		–0.0001 (–0.021 to 0.021)		0.99		0.003 (–0.076 to 0.081)		0.94
MnBP		–0.071 (–0.31 to 0.17)		0.56		–0.013 (–0.036 to 0.0010)		0.28		–0.029 (–0.11 to 0.056)		0.50
MCPP		–0.26 (–0.44 to –0.069)		0.007		–0.024 (–0.042 to –0.0057)		0.01		0.011 (–0.056 to 0.078)		0.74
BPA		–0.003 (–0.23 to 0.22)		0.98		0.0010 (–0.021 to 0.023)		0.93		0.053 (–0.027 to 0.13)		0.20
		Total T_3_ (ng/dL)				ln-Free T_3_ (pg/mL)				ln-Thyroglobulin (ng/mL)		
MEHP		2.41 (0.39 to 4.42)		0.02		0.0048 (–0.0046 to 0.014)		0.32		0.008 (–0.071 to 0.087)		0.84
MEHHP		2.56 (0.51 to 4.60)		0.01		0.0041 (–0.0055 to 0.014)		0.40		0.015 (–0.066 to 0.095)		0.72
MEOHP		2.76 (0.68 to 4.83)		0.009		0.0037 (–0.0061 to 0.013)		0.46		0.021 (–0.060 to 0.10)		0.61
MECPP		2.81 (0.48 to 5.14)		0.02		0.0052 (–0.0057 to 0.016)		0.35		0.020 (–0.071 to 0.11)		0.66
MiBP		2.30 (–0.81 to 5.42)		0.15		0.0083 (–0.0062 to 0.023)		0.26		–0.047 (–0.17 to 0.074)		0.45
MnBP		2.76 (–0.64 to 6.16)		0.11		0.0079 (–0.0079 to 0.024)		0.32		–0.007 (–0.14 to 0.13)		0.92
MCPP		0.61 (–2.06 to 3.28)		0.65		0.0022 (–0.010 to 0.015)		0.72		0.012 (–0.092 to 0.12)		0.82
BPA		0.43 (–2.78 to 3.64)		0.79		–0.0032 (–0.018 to 0.012)		0.67		–0.010 (–0.14 to 0.11)		0.87
**a**Adjusted for age, sex, race, BMI, ln-serum cotinine, ln-urinary creatinine, and ln-urinary iodine; 4 participants were missing data for BMI, 2 for serum cotinine, and 20 for urinary iodine.												

Results of the multivariable regression  analysis conducted among the adults (≥ 20 years of age; *n* = 1,346) are presented in Tables 1 and 2. In the analysis unweighted for sampling strategy (Table 1), we observed significant inverse relationships between all four DEHP metabolites (MEHP, MEHHP, MEOHP, and MECPP) and total T_4_. For example, for the median total T_4_ level among adults in this population (7.6 µg/mL), an IQR increase in MEHHP was associated with a 4.1% [95% confidence interval (CI), –5.9 to –2.6] decline in total T_4_. There was also an inverse association between MEHHP and free T_4_ and between MCPP and free T_3_. Urinary BPA was inversely related to total T_4_ as well, where an IQR increase in BPA was associated with a 1.7% (95% CI, –3.5 to –0.01) decline in total T_4_. In the results weighted for sampling strategy (Table 2), the significant inverse relationships remained between DEHP metabolites and total T_4_ and between MCPP and free T_3_. The coefficients were similar between modeling approaches but were less precise in the weighted models. The inverse relationships between DEHP metabolites and free T_4_ and between BPA and total T_4_ were weakened when the sample weights were included. However, several other significant associations emerged. There were larger (farther from the null) and statistically significant regression coefficients involving total T_3_, TSH, and thyroglobulin, where multiple DEHP metabolites were positively associated with TSH and inversely associated with total T_3_ and thyroglobulin. No significant relationships involving MiBP or MnBP were observed with either modeling approach.

**Figure 1:**
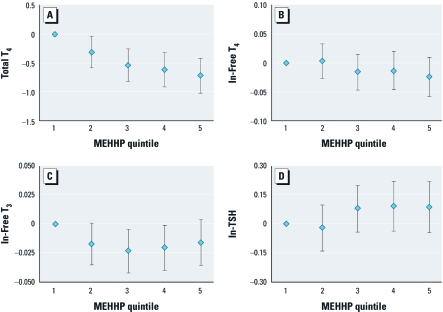
Adjusted regression coefficients [β (95% CI)] for change in serum thyroid measure in relation to urinary MEHHP quintiles (*p-*value for trend) for (*A*) total T_4_ (< 0.0001), (*B*) free T_4_ (0.09), (*C*) free T_3_ (0.20), and (*D*) TSH (0.06). Values are adjusted for age, sex, race, BMI, ln-serum cotinine, ln-urinary creatinine, and ln‑urinary iodine.

**Figure 2:**
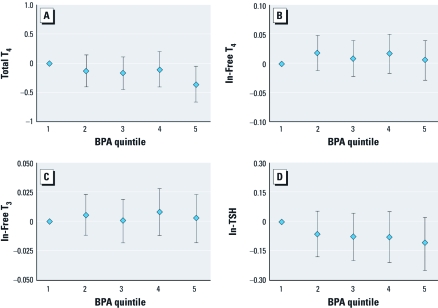
Adjusted regression coefficients [β (95% CI)] for change in serum thyroid measure in relation to urinary BPA quintiles (*p-*value for trend) for (*A*) total T_4_ (0.05), (*B*) free T_4_ (0.89), (*C*) free T_3_ (0.80), and (*D*) TSH (0.14). Values are adjusted for age, sex, race, BMI, ln-serum cotinine, ln-urinary creatinine, and ln-urinary iodine.

When the analysis of adults was stratified by sex, associations between urinary phthalate or BPA concentrations and free and total T_4_, total T_3_, and TSH were consistent between groups. The primary difference between males and females involved the regression coefficients for thyroglobulin. Significant or suggestive inverse associations (*p*-values ranging from 0.04 to 0.1) were observed between all four DEHP metabolites and thyroglobulin among females, whereas among males coefficients were positive and did not approach statistical significance (*p*-values ranged from 0.4 to 0.9; results not shown). When urinary BPA or phthalates were categorized into quintiles, we observed the same overall patterns. For example, Figures 1A–D show the relationship between MEHHP quintiles and total T_4_, free T_4_, total T_3_, and TSH, respectively. We observed a strong monotonic inverse relationship between MEHHP quintiles and total T_4_ (*p* for trend < 0.0001; Figure 1A). There was a suggestive inverse trend between MEHHP quintiles and free T_4_ (*p* for trend = 0.09; Figure 1B) and a suggestive positive trend between MEHHP quintiles and TSH (*p* for trend = 0.06; Figure 1D). There was evidence of a nonmonotonic relationship between MEHHP quintiles and free T_3_ (Figure 1C), where free T_3_ levels in the third and fourth quintiles were significantly reduced compared with the lowest MEHHP quintile. Results for MEOHP and MECCP (not shown) were similar to those for MEHHP. Relationships between urinary BPA quintiles and total T_4_, free T_4_, total T_3_, and TSH are presented in Figure 2A–D, respectively. We observed suggestive inverse trends for BPA quintiles and total T_4_ (*p* for trend = 0.05; Figure 2A) and TSH (*p* for trend = 0.14; Figure 2D).

Results of the multivariable regression analysis conducted among the adolescents (12–19 years of age) are presented in Table 3. The sample size available for analysis was much smaller than for the adults (*n* = 329). However, significant positive associations between DEHP metabolites and total T_3_ were observed in both the weighted [Supplemental Material, Table 4 (http://dx.doi.org/10.1289/ehp.1103582)] and unweighted (Table 3) models. An IQR increase in MEHHP was associated with a 3.8% (95% CI, 0.7 to 6.6) and 5.5% (95% CI, 2.1 to 9.0) increase in total T_3_ in the unweighted and weighted models, respectively, relative to the median T_3_ level among the adolescents (126 ng/dL). In the unweighted models (Table 3), the secondary DEHP metabolites were also positively associated with TSH; these relationships were weakened when sampling weights were included in the models (see Supplemental Material, Table 4). There were also significant inverse associations between MCPP and free and total T_4_ among this age group in both the weighted and unweighted analysis. When stratifying the analysis among adolescents by sex, the regression coefficients were of similar magnitude for both groups, with the exception of TSH, where the positive relationships with the secondary DEHP metabolites was stronger among females (data not shown).

## Discussion

In our analysis of data available from NHANES 2007–2008, we found consistent evidence for inverse relationships between urinary metabolites of DEHP and total T_4_ in adult participants. Depending on whether sampling weights were included in the models to account for the sampling design, we also found evidence for associations between DEHP metabolites and reduced total T_3_, free T_4_, and thyroglobulin, and increased TSH. These results were somewhat consistent with a previous study of 408 men recruited through a U.S. infertility clinic that reported inverse associations between urinary MEHP and serum levels of free T_4_ and total T_3_ (Meeker et al. 2007). However, no relationships were observed between phthalate metabolites and TSH in the previous study, and total T_4_ and free T_3_ were not measured. The present results are also consistent with limited animal studies reporting that rats fed DEHP through the diet displayed histopathologic alterations in the thyroid indicative of hyperactivity, increased thyroglobulin turnover, and significantly decreased plasma T_4_ levels (Hinton et al. 1986; Howarth et al. 2001; Poon et al. 1997; Price et al. 1988). More recently, *in vitro* studies have reported that DEHP acts as thyroid receptor antagonist (Ghisari and Bonefeld-Jorgensen 2009; Shen et al. 2009; Shi et al. 2011) and alters sodium-iodide symporter (NIS)–mediated iodide uptake (Wenzel et al. 2005). Although human and experimental studies on phthalates and thyroid function are currently limited, the results of those studies, along with the nature of the relationships we observed in the present study, may provide some general mechanistic clues. Our observation in adults that DEHP metabolites were inversely associated with thyroid hormones and thyroglobulin but positively associated with TSH suggest that DEHP exposure is associated with alterations in thyroid hormone synthesis, release, transport, or metabolism as opposed to effects on the hypothalamus or anterior pituitary. However, more detailed studies are needed to more precisely identify potential sites and mechanisms of action.

Results among participants 12–19 years of age were inconsistent with those from the adults. Although the sample size for this age group was considerably smaller, we found evidence of positive relationships between DEHP metabolites and T_3_ and TSH. As far as we are aware, associations between phthalates and thyroid hormone levels have not been explored in this age range in previous human studies. However, these findings are in contrast to a Danish study of 845 children (4–9 years of age) that reported inverse relationships between metabolites of DEHP (and other phthalates) and free and total T_3_ (Boas et al. 2010). Although differences in study populations and ages may account for these differences in association, differences in exposure levels between populations may also be involved. The median (75th percentile) MEHP concentration among adolescents in the present study was 2.0 (4.5) µg/g creatinine compared with 6.8 (11.0) µg/g creatinine in the Danish study. Alternatively, it is possible that the positive associations between DEHP metabolites and T_3_ among adolescents reported here could be a chance finding or due to residual confounding. For example, T_3_ levels are elevated in childhood until approximately 10 years of age and then decline gradually but significantly throughout adolescence until normal adult levels are reached (Corcoran et al. 1977). Although we adjusted for age and BMI in our models, the beginning of the decline in circulating T_3_ coincides with the onset of puberty (Corcoran et al. 1977). Adolescents at the ages studied here represent a wide range of developmental stages, and stages of development between individuals of the same age can vary greatly. Thus, within a certain age, more-developed children would be expected to have lower T_3_ levels than less-developed children of the same age. Further, like total T_3_, DEHP metabolite concentrations are inversely associated with age (CDC 2010; Silva et al. 2004). If these elevated phthalate concentrations among younger people are also due to developmental stage, where smaller and less-developed children with the same exposure to DEHP would experience a greater dose per body weight and excrete greater concentrations of DEHP metabolites (and/or lower concentrations of creatinine) in urine compared with larger and more developed children, there may have been residual confounding that resulted in the positive relationships observed. Future studies of adolescents should consider other anthropometric measures, as well as information on pubertal stage, when assessing relationships between urinary phthalate metabolites and thyroid hormone levels.

Urinary MCPP, an oxidized metabolite of both DBP and DOP, was associated with decreased thyroid hormones in several models among both adults and children. Although data on the potential effects of DOP on thyroid function in the literature are limited, high concentrations of DOP were associated with thyroid histological changes and reduced follicle size and colloid density in rats (Poon et al. 1997). DOP also up-regulated—while DBP down-regulated—sodium/iodide symporter (NIS) gene transcription in an *in vitro* study of human thyroid PCCL3 cells (Breous et al. 2005). DOP and DBP were also detected in the well water supply in an iodine-sufficient yet endemic goiter area of western Colombia (Gaitan 1983).

In the present study, we did not find evidence for associations between the primary DBP metabolites (MnBP and MiBP) and serum thyroid markers among adults or adolescents in NHANES 2007–2008. This is consistent with the previous study of adult men attending an infertility clinic (Meeker et al. 2007) but inconsistent with a smaller study of pregnant women (*n* = 76) in which statistically significant inverse relationships were reported between urinary MBP and serum levels of free and total T_4_ (Huang et al. 2007). In addition to an inverse relationship between urinary DEHP metabolites and free and total T_3_ reported in Danish children described above, Boas et al. (2010) also reported inverse relationships between urinary MBP and total T_3_ among girls and between MBP and free T_3_ among boys. One potential explanation for these inconsistencies could be the differences in exposure levels between the populations. For example, the median (75th percentile) urinary MBP (MnBP + MiBP) concentrations in the Danish children were 191 (276) and 227 (312) µg/g creatinine for boys and girls, respectively (Boas et al. 2010), compared with 30.1 (49.5) µg/g creatinine in the present study. Likewise, the median (75th percentile) urinary MBP concentration in the study of Taiwanese pregnant women was 195 (339) µg/g creatinine.

We observed a suggestive inverse relationship between BPA and total T_4_ among the adults in our analysis. In a recent study of men recruited through an infertility clinic, BPA was inversely associated with TSH but not related to free T_4_ or total T_3_ (Meeker et al. 2010). In the present study, during the course of our preliminary analysis we found a significant inverse association between BPA and TSH (results not shown). However, upon carrying out regression diagnostics we found that one of the three outliers that we eventually removed from our final models was highly influential, and the association was no longer significant when the outliers were not included in the analysis. Limited and inconsistent studies suggest BPA or its halogenated derivatives may interact as an agonist or antagonist on the thyroid receptor (Diamanti-Kandarakis et al. 2009; Heimeier et al. 2009; Moriyama et al. 2002). Potentially consistent with our observation of suggestive inverse associations between BPA and both T_4_ and TSH, a study by Kaneko et al. (2008) reported that BPA suppresses TSH release from amphibian pituitary in a manner independent of both the thyroid hormone feedback mechanism and the estrogenic activity of BPA. These studies and the present analysis suggest that BPA may alter thyroid signaling, but more research is needed.

There were several limitations to the present analysis. First, because of the observational and cross-sectional study design, we are unable to make any conclusions regarding causation in the relationships between BPA or phthalate exposure and thyroid measures. In addition, only one urine sample per subject was analyzed for BPA and phthalate metabolite concentrations, which may not be representative of the average body burden of the subject, because these chemicals are metabolized rapidly. The intraclass correlation coefficient (ICC) for phthalates in repeated urine samples collected over weeks or months ranges from 0.2 to 0.7 and differs by metabolite (Hauser et al. 2004; Peck et al. 2010; Suzuki et al. 2009; Teitelbaum et al. 2008). The ICC for BPA is likely even lower, between 0.1 and 0.3 (Mahalingaiah et al. 2008; Meeker et al. 2011; Teitelbaum et al. 2008). It is possible that the higher temporal variability in urinary BPA led to increased exposure measurement error and weaker effect estimates in relation to thyroid measures. However, previous studies have demonstrated that single spot urine samples of phthalate or BPA concentrations may be moderately representative of long-term averages when categorized into broad exposure groups (e.g., quintiles) (Hauser et al. 2004, Mahalingaiah et al. 2008). The data set was also limited to serum thyroid measures collected at a single time point for each participant, although Andersen et al. (2002) demonstrated that thyroid function measures within an individual are maintained within relatively narrow limits over time.

## Conclusions

Overall, this analysis of a representative sample of U.S. adults and adolescents supports the previous reports of a relationship between urinary phthalate metabolites and serum thyroid hormone levels. We also found suggestive evidence for relationships involving BPA. More detailed studies of populations at various life stages are needed to reconcile the specific findings of the limited human and animal research conducted in this area to date, establish the temporal relationships between markers of exposure and effect, elucidate the biologic mechanism(s) involved, and determine the potential clinical and public health implications of these associations.

## Supplemental Material

(44 KB) PDF

## References

[r1] Andersen S, Pedersen KM, Bruun NH, Laurberg P (2002). Narrow individual variations in serum T_4_ and T_3_ in normal subjects: a clue to the understanding of subclinical thyroid disease.. J Clin Endocrinol Metab.

[r2] Barr DB, Wilder LC, Caudill SP, Gonzalez AJ, Needham LL, Pirkle JL (2005). Urinary creatinine concentrations in the U.S. population: implications for urinary biologic monitoring measurements.. Environ Health Perspect.

[r3] Boas M, Feldt-Rasmussen U, Skakkebaek NE, Main KM (2006). Environmental chemicals and thyroid function.. Eur J Endocrinol.

[r4] Boas M, Frederiksen H, Feldt-Rasmussen U, Skakkebaek NE, Hegedus L, Hilsted L (2010). Childhood exposure to phthalates: associations with thyroid function, insulin-like growth factor I, and growth.. Environ Health Perspect.

[r5] Braun JM, Yolton K, Dietrich KN, Hornung R, Ye X, Calafat AM (2009). Prenatal bisphenol A exposure and early childhood behavior.. Environ Health Perspect.

[r6] Breous E, Wenzel A, Loos U. (2005). The promoter of the human sodium/iodide symporter responds to certain phthalate plasticisers.. Mol Cell Endocrinol.

[r7] Calafat AM, Ye X, Wong LY, Reidy JA, Needham LL (2008). Exposure of the U.S. population to bisphenol A and 4-*tertiary*-octylphenol: 2003–2004.. Environ Health Perspect.

[r8] CDC (Centers for Disease Control and Prevention) (2010). Fourth National Report on Human Exposure to Environmental Chemicals.

[r9] Cho SC, Bhang SY, Hong YC, Shin MS, Kim BN, Kim JW (2010). Relationship between environmental phthalate exposure and the intelligence of school-age children.. Environ Health Perspect.

[r10] Corcoran JM, Eastman CJ, Carter JN, Lazarus L (1977). Circulating thyroid hormone levels in children.. Arch Dis Child.

[r11] Diamanti-Kandarakis E, Bourguignon JP, Giudice LC, Hauser R, Prins GS, Soto AM (2009). Endocrine-disrupting chemicals: an Endocrine Society scientific statement.. Endocr Rev.

[r12] Engel SM, Miodovnik A, Canfield RL, Zhu C, Silva MJ, Calafat AM (2010). Prenatal phthalate exposure is associated with childhood behavior and executive functioning.. Environ Health Perspect.

[r13] Engel SM, Zhu C, Berkowitz GS, Calafat AM, Silva MJ, Miodovnik A (2009). Prenatal phthalate exposure and performance on the Neonatal Behavioral Assessment Scale in a multiethnic birth cohort.. Neurotoxicology.

[r14] Gaitan E. (1983). Endemic goiter in western Colombia.. Ecol Dis.

[r15] Galloway T, Cipelli R, Guralnick J, Ferrucci L, Bandinelli S, Corsi AM (2010). Daily bisphenol A excretion and associations with sex hormone concentrations: results from the InCHIANTI Adult Population Study.. Environ Health Perspect.

[r16] Ghisari M, Bonefeld-Jorgensen EC (2009). Effects of plasticizers and their mixtures on estrogen receptor and thyroid hormone functions.. Toxicol Lett.

[r17] Hatch EE, Nelson JW, Stahlhut RW, Webster TF (2010). Association of endocrine disruptors and obesity: perspectives from epidemiological studies.. Int J Androl.

[r18] Hauser R, Meeker JD, Park S, Silva MJ, Calafat AM (2004). Temporal variability of urinary phthalate metabolite levels in men of reproductive age.. Environ Health Perspect.

[r19] Heimeier RA, Das B, Buchholz DR, Shi YB (2009). The xenoestrogen bisphenol A inhibits postembryonic vertebrate development by antagonizing gene regulation by thyroid hormone.. Endocrinology.

[r20] Hinton RH, Mitchell FE, Mann A, Chescoe D, Price SC, Nunn A (1986). Effects of phthalic acid esters on the liver and thyroid.. Environ Health Perspect.

[r21] Hornung RW, Reed L (1990). Estimation of average concentration in the presence of nondetectable values.. Appl Occup Environ Hyg.

[r22] Howarth JA, Price SC, Dobrota M, Kentish PA, Hinton RH (2001). Effects on male rats of di-(2-ethylhexyl) phthalate and di-n-hexylphthalate administered alone or in combination.. Toxicol Lett.

[r23] Huang PC, Kuo PL, Guo YL, Liao PC, Lee CC (2007). Associations between urinary phthalate monoesters and thyroid hormones in pregnant women.. Hum Reprod.

[r24] Kaneko M, Okada R, Yamamoto K, Nakamura M, Mosconi G, Polzonetti-Magni AM (2008). Bisphenol A acts differently from and independently of thyroid hormone in suppressing thyrotropin release from the bullfrog pituitary.. Gen Comp Endocrinol.

[r25] Kim BN, Cho SC, Kim Y, Shin MS, Yoo HJ, Kim JW (2009). Phthalates exposure and attention-deficit/hyperactivity disorder in school-age children.. Biol Psychiatry.

[r26] Korn EL, Graubard BI (1991). Epidemiologic studies utilizing surveys: accounting for the sampling design.. Am J Public Health.

[r27] Lang IA, Galloway TS, Scarlett A, Henley WE, Depledge M, Wallace RB (2008). Association of urinary bisphenol A concentration with medical disorders and laboratory abnormalities in adults.. JAMA.

[r28] Mahalingaiah S, Meeker JD, Pearson KR, Calafat AM, Ye X, Petrozza J (2008). Temporal variability and predictors of urinary bisphenol A concentrations in men and women.. Environ Health Perspect.

[r29] Meeker JD, Calafat AM, Hauser R (2007). Di(2-ethylhexyl) phthalate metabolites may alter thyroid hormone levels in men.. Environ Health Perspect.

[r30] Meeker JD, Calafat AM, Hauser R (2010). Urinary bisphenol A concentrations in relation to serum thyroid and reproductive hormone levels in men from an infertility clinic.. Environ Sci Technol.

[r31] Meeker JD, Yang T, Ye X, Calafat AM, Hauser R (2011). Urinary concentrations of parabens and serum hormone levels, semen quality parameters, and sperm DNA damage.. Environ Health Perspect.

[r32] Miller MD, Crofton KM, Rice DC, Zoeller RT (2009). Thyroid-disrupting chemicals: interpreting upstream biomarkers of adverse outcomes.. Environ Health Perspect.

[r33] Moriyama K, Tagami T, Akamizu T, Usui T, Saijo M, Kanamoto N (2002). Thyroid hormone action is disrupted by bisphenol A as an antagonist.. J Clin Endocrinol Metab.

[r34] NCHS (National Center for Health Statistics) (2009). 2007–2008 Documentation, Codebook, and Frequencies for Thyroid Profile.. http://www.cdc.gov/nchs/nhanes/nhanes2007-2008/THYROD_E.htm.

[r35] NCHS (National Center for Health Statistics) (2010a). Continuous NHANES Web Tutorial Home.. http://www.cdc.gov/nchs/tutorials/Nhanes/index_current.htm.

[r36] NCHS (National Center for Health Statistics) (2010b). National Health and Nutrition Examination Survey.. http://www.cdc.gov/nchs/nhanes.htm.

[r37] NCHS (National Center for Health Statistics) (2010c). 2007–2008 Data Documentation, Codebook, and Frequencies: Environmental Phenols (EPH_E).. http://www.cdc.gov/nchs/nhanes/nhanes2007-2008/EPH_E.htm.

[r38] NCHS (National Center for Health Statistics) (2010d). 2007–2008 Data Documentation, Codebook, and Frequencies: Urinary Phthalates (PHTHTE_E).. http://www.cdc.gov/nchs/nhanes/nhanes2007-2008/PHTHTE_E.htm.

[r39] O’Connor JC, Frame SR, Ladics GS (2002). Evaluation of a 15-day screening assay using intact male rats for identifying antiandrogens.. Toxicol Sci.

[r40] Peck JD, Sweeney AM, Symanski E, Gardiner J, Silva MJ, Calafat AM (2010). Intra- and inter-individual variability of urinary phthalate metabolite concentrations in Hmong women of reproductive age.. J Expo Sci Environ Epidemiol.

[r41] Poon R, Lecavalier P, Mueller R, Valli VE, Procter BG, Chu I (1997). Subchronic oral toxicity of di-n-octyl phthalate and di(2-ethylhexyl) phthalate in the rat.. Food Chem Toxicol.

[r42] Price SC, Chescoe D, Grasso P, Wright M, Hinton RH (1988). Alterations in the thyroids of rats treated for long periods with di-(2-ethylhexyl) phthalate or with hypolipidaemic agents.. Toxicol Lett.

[r43] Shen O, Du G, Sun H, Wu W, Jiang Y, Song L (2009). Comparison of *in vitro* hormone activities of selected phthalates using reporter gene assays.. Toxicol Lett.

[r44] Shi W, Wang X, Hu G, Hao Y, Zhang X, Liu H (2011). Bioanalytical and instrumental analysis of thyroid hormone disrupting compounds in water sources along the Yangtze River.. Environ Pollut.

[r45] Silva MJ, Barr DB, Reidy JA, Malek NA, Hodge CC, Caudill SP (2004). Urinary levels of seven phthalate metabolites in the U.S. population from the National Health and Nutrition Examination Survey (NHANES) 1999–2000.. Environ Health Perspect.

[r46] Suzuki Y, Niwa M, Yoshinaga J, Watanabe C, Mizumoto Y, Serizawa S (2009). Exposure assessment of phthalate esters in Japanese pregnant women by using urinary metabolite analysis.. Environ Health Prev Med.

[r47] Teitelbaum SL, Britton JA, Calafat AM, Ye X, Silva MJ, Reidy JA (2008). Temporal variability in urinary concentrations of phthalate metabolites, phytoestrogens and phenols among minority children in the United States.. Environ Res.

[r48] Wenzel A, Franz C, Breous E, Loos U. (2005). Modulation of iodide uptake by dialkyl phthalate plasticisers in FRTL-5 rat thyroid follicular cells.. Mol Cell Endocrinol.

[r49] Westgard JO, Barry PL, Hunt MR, Groth T (1981). A multi-rule Shewhart chart for quality control in clinical chemistry.. Clin Chem.

[r50] Wetherill YB, Akingbemi BT, Kanno J, McLachlan JA, Nadal A, Sonnenschein C (2007). *In vitro* molecular mechanisms of bisphenol A action.. Reprod Toxicol.

[r51] Ye X, Kuklenyik Z, Needham LL, Calafat AM (2005). Automated on-line column-switching HPLC-MS/MS method with peak focusing for the determination of nine environmental phenols in urine.. Anal Chem.

[r52] Zoeller RT (2007). Environmental chemicals impacting the thyroid: targets and consequences.. Thyroid.

