# Supplementary material for: Relationship between Urinary Phthalate and Bisphenol A Concentrations and Serum Thyroid Measures in U.S. Adults and Adolescents from the National Health and Nutrition Examination Survey (NHANES) 2007–2008
Source: Environ Health Perspect. 2011 Jul 11;119(10):1396–402. doi: 10.1289/ehp.1103582 (PMC3230451; doi:10.1289/ehp.1103582)
Supplement: (44 KB) PDF [file ehp.1103582.s001.pdf]

Supplemental Material

Relationship between Urinary Phthalate and Bisphenol A Concentrations and Serum Thyroid Measures in U.S. Adults and Adolescents from NHANES 2007-08

John D. Meeker[1], Kelly K. Ferguson[1]

[1]Department of Environmental Health Sciences, University of Michigan School of Public Health, Ann Arbor, MI

Table of Contents:

Page 2: Supplemental Material, Table 1. Study population characteristics for adolescents and adults, with and without sample weights, NHANES 2007-08.

Page 3: Supplemental Material, Table 2. Creatinine-corrected urinary phthalate metabolite concentrations, NHANES 2007-08 (µg/g creatinine).

Page 4: Supplemental Material, Table 3. Distribution of thyroid measures, NHANES 2007-08.

Page 5: Supplemental Material, Table 4. Adjusted regression coefficients (95% confidence intervals) for change in serum thyroid measure in relation to a unit increase in ln-transformed urinary phthalate or BPA concentration among adolescents (ages 12-19). Results weighted for sampling strategy.

Supplemental Material, Table 1. Study population characteristics for adolescents and adults, with and without sample weights, NHANES 2007-08.

Variable	Category	Adults (ages >=20; N = 1405)			Adolescents (ages 12-19; N = 355)		
		N	% Unweighted[a]	% Weighted[b]	N	% Unweighted[a]	% Weighted[b]
Gender	Male	737	52.5	52.5	185	52.1	53.4
	Female	668	47.5	47.5	170	47.9	46.6
Race/ethnicity	Non-Hispanic White	643	45.8	69.7	117	33.0	63.0
	Non-Hispanic Black	280	19.9	10.7	90	23.4	14.0
	Mexican American	270	19.2	9.2	82	23.1	11.3
	Other Hispanic	158	11.3	5.0	51	14.4	7.3
	Other race/multi-racial	54	3.8	5.4	15	4.2	4.5
BMI (kg/m^2)	Underweight (<18.5)	16	1.2	1.0	50	14.3	14.1
	Normal weight (18.5-24.9)	384	27.5	32.0	176	50.1	53.7
	Pre-obese (25-29.9)	468	33.6	32.3	74	21.1	19.3
	Obese class I (30-34.9)	286	20.5	19.4	26	7.4	7.6
	Obese class II and III (>34.9)	241	17.3	15.3	25	7.1	5.3

[a]unweighted represents actual sample percentages

[b]weighted represents population percentages

Supplemental Material, Table 2. Creatinine-corrected urinary phthalate metabolite concentrations, NHANES 2007-08 (µg/g creatinine).

Age Group	Urinary Analyte	% < LOD	Geometric Mean	Selected Percentiles					
				25th	50th	75th	90th	95th	Max.
Adults (ages >=20)	MEHP	33.7	2.63	<LOD	2.29	5.20	11.8	21.3	890
	MEHHP	0.9	20.6	9.84	18.23	37.0	89.6	167	3135
	MEOHP	2.0	11.2	5.43	9.76	20.5	43.4	88.6	1641
	MECPP	0.1	30.6	15.4	26.4	50.8	121	232	5808
	MiBP	2.3	6.63	4.17	6.67	11.1	17.0	24.1	159
	MnBP	0.9	17.5	10.4	17.1	28.1	45.9	69.9	2613
	MCPP	3.1	2.42	1.35	2.26	3.95	7.71	11.5	157
	BPA	7.1	2.03	1.17	1.92	3.33	6.02	8.87	149
Adolescents (ages 12-19)	MEHP	28.7	2.38	<LOD	2.00	4.50	13.3	27.4	402
	MEHHP	0	23.5	10.3	20.33	45.32	157.91	254	1400
	MEOHP	0	13.31	5.79	11.44	24.74	86.27	132	695
	MECPP	0	34.72	16.7	27.8	64.8	164	265	1099
	MiBP	0.6	8.01	4.69	8.24	13.73	21.61	28.78	91.2
	MnBP	0.3	20.65	12.37	21.93	35.86	52.75	72.54	240.54
	MCPP	1.7	3.01	1.67	2.93	5.16	8.81	11.25	909.68
	BPA	2.8	1.88	1.10	1.67	2.93	5.02	6.88	49.5

Supplemental Material, Table 3. Distribution of thyroid measures, NHANES 2007-08.

Age Group	Thyroid measure	Units	Geometric Mean	25th percentile	Median	75th percentile	Reference Range[a]	% below range	% above range
Adults									
(ages >=20)	Total T4	ug/mL	7.7[b]	6.7	7.6	8.5	4-11	0.2	2.7
	Free T4	ng/dL	0.8	0.7	0.8	0.9	0.7-2.1	15.6	0
	Total T3	ng/dL	113[b]	98	111	124	75-175	2.3	1.6
	Free T3	pg/mL	3.2	2.9	3.2	3.4	2-5	0	0.1
	TSH	uIU/mL	1.6	1.1	1.6	2.3	0.3-4	1.1	5.8
	Thyroglobulin	ng/mL	10.2	6.2	10.4	18.7	1-20	2.8	22.1
Adolescents									
(ages 12-19)	Total T4	ug/mL	7.6[b]	6.6	7.3	8.4	4-11	0	2.8
	Free T4	ng/dL	0.8	0.7	0.8	0.9	0.7-2.1	13.5	0
	Total T3	ng/dL	129[b]	112	126	144	N/A[c]	-	-
	Free T3	pg/mL	3.6	3.3	3.6	3.9	N/A[c]	-	-
	TSH	uIU/mL	1.4	1.0	1.4	2.1	0.3-4	0.9	2.5
	Thyroglobulin	ng/mL	8.1	5.6	9.2	14.5	1-20	3.7	8.5

[a]De Groot LJ, Hennemann G. 2010. Thyroid Disease Manager, Chapter 6b: Clinical Strategies in the Testing of Thyroid Function. Available: http://www.thyroidmanager.org/Chapter6a/6a-frame.htm [accessed 4 April 2011].

[b]Arithmetic mean

[c]Not available; reference ranges for total and free T3 expected to be higher in children than adults

Supplemental Material, Table 4. Adjusted[a] regression coefficients (95% confidence intervals) for change in serum thyroid measure in relation to a unit increase in ln-transformed urinary phthalate or BPA concentration among adolescents (ages 12-19). Results weighted for sampling strategy. N = 329[b].

Analyte	Total T4 (µg/mL)		ln-Free T4 (ng/dL)		ln-TSH (uIU/mL)	
	β (95% CI)	p-value	β (95% CI)	p-value	β (95% CI)	p-value
MEHP	0.11 (-0.10, 0.33)	0.28	-0.0060 (-0.025, 0.013)	0.52	-0.004 (-0.055, 0.046)	0.86
MEHHP	0.089 (-0.11, 0.29)	0.36	0.0003 (-0.017, 0.018)	0.97	0.045 (-0.026, 0.12)	0.20
MEOHP	0.094 (-0.099, 0.29)	0.32	-0.0007 (-0.018, 0.017)	0.94	0.048 (-0.028, 0.12)	0.20
MECPP	0.15 (-0.046, 0.35)	0.12	0.0033 (-0.018, 0.024)	0.75	0.048 (-0.029, 0.13)	0.20
MiBP	-0.039 (-0.25, 0.17)	0.70	-0.0018 (-0.029, 0.026)	0.89	-0.011 (-0.12, 0.097)	0.83
MnBP	-0.044 (-0.35, 0.26)	0.77	-0.021 (-0.047, 0.0056)	0.11	-0.041 (-0.17, 0.086)	0.51
MCPP	-0.24 (-0.38, -0.094)	0.003	-0.023 (-0.043, -0.0019)	0.03	-0.010 (-0.096, 0.076)	0.81
BPA	-0.073 (-0.44, 0.30)	0.68	-0.0022 (-0.020, 0.016)	0.80	0.048 (-0.023, 0.12)	0.17
	Total T3 (ng/dL)		ln-Free T3 (pg/mL)		ln-Thyroglobulin (ng/mL)	
	β (95% CI)	p-value	β (95% CI)	p-value	β (95% CI)	p-value
MEHP	4.00 (1.97, 6.03)	0.0007	0.0081 (0.00035, 0.016)	0.04	-0.026 (-0.12, 0.065)	0.56
MEHHP	3.85 (1.44, 6.27)	0.004	0.0084 (-0.0026, 0.019)	0.12	-0.026 (-0.13, 0.074)	0.59
MEOHP	4.24 (1.72, 6.75)	0.003	0.0082 (-0.0036, 0.020)	0.16	-0.024 (-0.13, 0.081)	0.63
MECPP	4.70 (1.97, 7.43)	0.002	0.011 (-0.00096, 0.023)	0.07	-0.024 (-0.14, 0.089)	0.66
MiBP	3.00 (-0.36, 6.37)	0.08	0.015 (-0.0016, 0.031)	0.07	-0.12 (-0.27, 0.019)	0.08
MnBP	2.42 (-3.17, 8.02)	0.37	0.014 (-0.0059, 0.034)	0.16	-0.087 (-0.22, 0.050)	0.20
MCPP	1.88 (-0.99, 4.75)	0.18	0.0055 (-0.0081, 0.019)	0.40	-0.031 (-0.23, 0.17)	0.74
BPA	1.46 (-4.53, 7.45)	0.61	0.0014 (-0.019, 0.021)	0.89	-0.095 (-0.35, 0.16)	0.44

[a]Adjusted for age, sex, race, BMI, ln-serum cotinine, ln-urinary creatinine, and ln-urinary iodine.

[b]4 Participants were missing data for BMI, 2 for serum cotinine, and 20 for urinary iodine.
